# Cross-cultural adaptation of the CRS-PRO questionnaire into French

**DOI:** 10.1186/s40463-023-00683-0

**Published:** 2023-12-09

**Authors:** Maxime Fieux, Florent Carsuzaa, Jeremy Charriot, Justin Michel, Fabien Subtil, Leigh J. Sowerby, Thomas Radulesco, Valentin Favier

**Affiliations:** 1 Service d’ORL, d’otoneurochirurgie et de chirurgie cervico-faciale, Centre Hospitalier Lyon Sud, Hospices Civils de Lyon, 69495 Pierre-Bénite Cedex, France; 2 Université de Lyon, Université Lyon 1, 69003 Lyon, France; 3 Univ Paris Est Creteil, INSERM, IMRB, 94010 Créteil, France; 4 CNRS EMR 7000, 94010 Créteil, France; 5 Department of Otorhinolaryngology – Head and neck surgery, University Hospital of Poitiers, Poitiers, France; 6 Service de Pneumologie, Centre Hospitalo-Universitaire de Montpellier, 80 Avenue Augustin Fliche, 34000 Montpellier, France; 7 ENT ‐ Head and Neck Surgery Department, APHM, La Conception University Hospital, Aix Marseille University, Marseille, France; 8 CNRS, IUSTI, Aix Marseille University, Marseille, France; 9 Service de Biostatistique et Bioinformatique, Hospices Civils de Lyon, Lyon, France; 10 Laboratoire de Biométrie et Biologie Évolutive, CNRS, UMR 5558, Villeurbanne, France; 11 Department of Otolaryngology, University of Western Ontario, London, ON Canada; 12 Service d’ORL et de chirurgie cervico-faciale, Centre Hospitalo-Universitaire de Montpellier, 80 Avenue Augustin Fliche, 34000 Montpellier, France

**Keywords:** Cross-cultural, Chronic rhinosinusitis, Questionnaire, French

## Abstract

**Background:**

Chronic rhinosinusitis (CRS), encompasses many different clinical patterns with variable response to treatment. Precise criteria specifying disease severity and control are lacking in the current literature. Our aim was to perform a cross-cultural adaptation of the CRS-PRO, creating a French version for use as a routine questionnaire in the assessment of patients with CRS.

**Methods:**

The CRS-PRO questionnaire was translated according to the recommendations of the International Society for Pharmacoeconomics and Outcomes Research (ISPOR) through a three-step procedure including a backward translation.

**Results:**

Seven of 12 items were initially discordant between the three translators before achieving consensus (Step 1). Two of 12 items were discordant between the backward translation and the initial CRS-PRO version regarding the word “mucus”(Step 2). Step 3 allowed the creation of a French proof-read version of the CRS-PRO questionnaire. Thirty patients were included for initial validation, mean age of 49.2 ± 15 years and 63.3% (19/30) male. It took them 67 ± 23 s to complete the questionnaire without any patients requiring more than 2 min.

**Conclusion:**

This study presents the French version of the CRS-PRO questionnaire—an adapted, validated, and well-accepted instrument to evaluate the CRS symptoms in the French speaking population.

## Background

Chronic rhinosinusitis with nasal polyps (CRSwNP), encompasses many different clinical patterns with variable response to treatment [1]. Precise criteria defining disease severity and control are lacking in current literature [2].

While ear, nose and throat specialists (ENTs) are expanding the therapeutic options in CRSwNP, patient-reported outcome measures can be heterogenous. While the most widely used instrument in clinical trials is the Sinonasal Outcome Test with 22 items (SNOT-22), some rhinologists would counter that eustachian tube dysfunction is a documented association with CRS and the questionnaire can be confusing and takes too long to complete in a busy clinical practice [3]. To date, there is no assessment taking into account all the main functional, physical, biological and radiological signs, and their impact on QoL to establish the severity of CRSwNP and symptom evolution under treatment [4].

The recently developed 12-item Patient Reported Outcomes in Chronic Rhinosinusitis (CRS-PRO) is a patient-reported outcome measure with extensive documented input from patients diagnosed with both CRSwNP and CRSsNP. It has been validated and is found to be responsive to both medical and surgical therapy. It is a useful tool, easy to apply, with good validity in the evaluation of CRS symptoms as described by Lin K. A. et al. The shorter CRS-PRO has demonstrated better correlation with radiographic changes after medical management or endoscopic sinus surgery (ESS) when compared with the longer SNOT-22 [5–7]. The CRS-PRO is separated in 3 distinct subdomains: rhino-psychologic, facial discomfort, and cough [7].

Adaptation of questionnaires to other languages (an important proof of their validity and their international impact) makes it possible to ensure their conceptual equivalence with the original questionnaire. This study presents the cross-cultural adaptation process of the original CRS-PRO into its French version to be used as a routine questionnaire in the assessment of patients with CRSwNP and CRSsNP.

## Methods

The CRS-PRO is owned and copyrighted by, and the intellectual property of, Bruce K. Tan, MD, MS. Permission was obtained to use the CRS-PRO questionnaire from the development team. All patients who participated in the development gave their consent before participating in this study, which was carried out in accordance with the Declaration of Helsinki. All data were anonymized, and no identifying data were stored. The source language (original language in which the questionnaire was developed) was U.S. English. The target language (foreign language in which the questionnaire needed to be translated) was French. The CRS-PRO questionnaire was translated according to the recommendations of the International Society for Pharmacoeconomics and Outcomes Research (ISPOR) [8]. Two professional translators, who were native speakers of the source language and bilingual in the target language were recruited, as were QoL experts in the target language. Three rhinologists in the target language were also involved. The three-step procedure translation process was as follows:

*Step 1 (Forward translation)*: Three independent rhinologists produced a forward translation from the source language (English) into the target language (French). All were native speakers of French and spoke English fluently. They all discussed the translations and agreed upon a single reconciled version (the combined version). The aim was to achieve a conceptually equivalent translation of the original questionnaire; the language used had to be colloquial and easy to understand. By combining the three translations, we obtained *French version 1.*

*Step 2 (Backward translation)*: *French version 1* was translated into English by two native speakers of English who spoke French fluently. Indeed, this step requires a native speaker of the source language and bilingual in the target language who had no access to the original source version of the questionnaire. Items containing discrepancies were re-translated. Backward translation and a report of the translation were submitted to Dr. Tan for review and comment. This version was compared with the original CRS-PRO questionnaire and revised until a satisfactory translation was produced. Comparison of the backward version was made with the original source version to detect any misunderstandings, mistranslations, or inaccuracies in the intermediary forward version of the questionnaire. The development of a consensus and the translation by means of this committee methodology reduced the cultural and social bias that may result when only one or two translators are responsible for the translation. We thus obtained *French version 2.* This version was submitted to a panel of six health care professionals (5 ORL and 1 pneumologist) and 3 patients to determine whether it was understandable and easy to use. Further corrections led to the production of *French version 3.*

*Step 3 (Patient testing)*: *French version 3* was tested on 30 patients in 3 different French regions through face-to-face interviews, to determine whether instructions, filling method and items were understandable and unambiguous (men and women). Patients were all native speakers of the target language and of the appropriate age-group for the CRS-PRO questionnaire. The prevalence of CRSwNP increased with age in adults (≥ 18 years of age), particularly after 40 years of age eventhough CRSsNP was more prevalent in subjects younger than 40 years [9]. The appropriate age-group was determined as a mean over 40 years of age to include all CRS. Patients were asked to complete the questionnaire in the presence of a practitioner who noted their reactions regarding the understandability of each item. The number of subjects interviewed, their age, the time it took to complete the questionnaire, the difficulties encountered, the solutions suggested and retained and how the third version of the questionnaire was produced were collected. Thanks to these remarks, we modified the questionnaire and obtained *French version 4.* This version was proof-read leading to *French version 5.*

Before initiating the study, all researchers were trained in patient interviewing. Oral consent was obtained from all participants. As all questionnaires were completed, there were no concerns regarding missing data.

### Statistical analysis

We used questionnaires completed by patients and stored results in a secure database. To test the reproducibility of this new scale, test‐retest reliability was assessed in 10 patients. Patients answered the questionnaire again under the same conditions 7 days later. Consistency between responses was evaluated using intra-class correlation coefficient. The closer the coefficient to 1, the higher the repeatability. The statistical analyses were performed using R software (v. 1.3.10703, R Foundation for Statistical Computing, Vienna, Austria, www.r-project.org). P-values were calculated using two-sided tests.

## Results

**Fig. 1:**
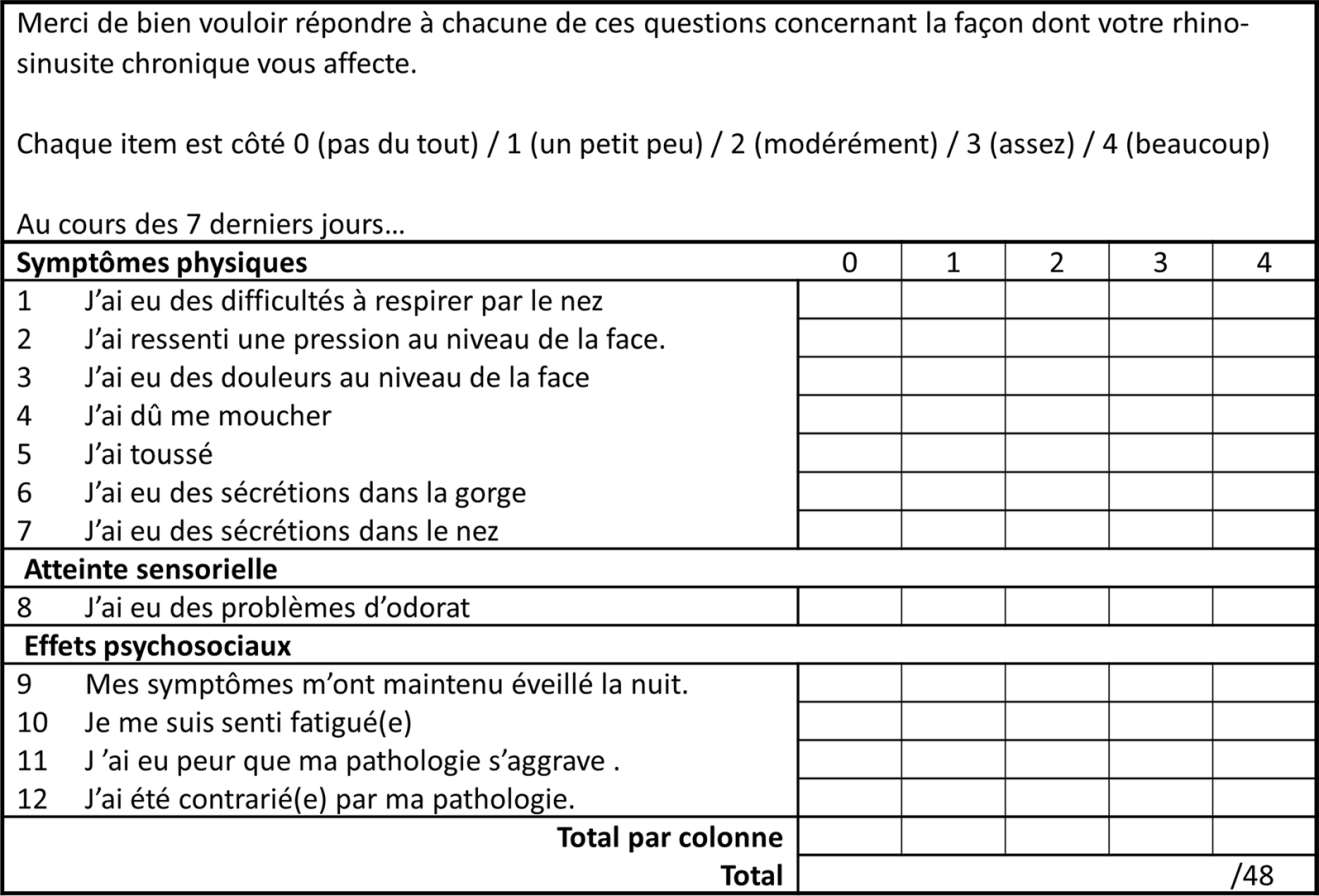
CRS-PRO French version 5

Seven of 12 items were initially discordant between the three translators before achieving a consensual version. Herein some examples: the word “face” was translated by “face” by two translators and “visage” by the other one, the translators settled for “face”; one of the three translators chose to use the present tense, but past was chosen to respect the English original version; and, for item 8, either “problème” or “trouble” was chosen by translators for the word “problems” and they settled for “problèmes” to be as accurate as possible (Step 1). 2 of 12 items were discordant between the backward translation and the initial CRS-PRO version: « I’ve felt pain in my face” instead of “my face hurt» and « I’ve been bothered by my state of health” instead of “I was frustrated by my condition», respectively. Accordingly, item 3 was modified into “J’ai eu des douleurs au niveau de la face” and item 12 into “J’ai été contrarié(e) par ma pathologie”. *French version 2* was then submitted to the panel. One item was modified according to healthcare professionals and patient’s feedback regarding the word “mucus” in English which can be translated into French either by “sécrétions” or “mucosités”. The word “sécrétions” was chosen leading to *French version 3*. Minor adaptations were done to obtain *French version 4* (Step 3). The definitive proof-read version (*French version 5*) is presented in Fig. 1.

Thirty patients were included, mean age of 49.2 ± 15 years old, with 50% of patients under 50 years old and 63.3% (19/30) male. It took them 67 ± 23 s to complete the full questionnaire without any patients requiring more than 2 min. Items as described in Fig. 1 were rated between 0 and 4 by all participants. No significant difference was observed between male and female except for item 7 and 9 (*p* = 0.009, and *p* = 0.008, respectively; Table 1). No significant difference was observed between age categories for all items (< 50 or 50 years old). The test-rest reliability was 0.98.

**Table 1:** Comparison of responses between Male and Female participants

	Female (n = 11)	Male (n = 19)	*p*-Value
General data			
Age (years)	49 ± 16.4	49.5 ± 14.4	0.97
Time to complete (seconds)	64 ± 29	69 ± 20	0.38
Answers to the CRS-PRO			
Item 1 “nasal breathing”			
0	1 (9.1%)	4 (21.1%)	0.25
1	4 (36.4%)	4 (21.1%)	
2	5 (45.5%)	4 (21.1%)	
3	0 (0.0%)	5 (26.3%)	
4	1 (9.1%)	2 (10.5%)	
Item 2 “pressure”			
0	3 (27.3%)	9 (47.4%)	0.56
1	2 (18.2%)	4 (21.1%)	
2	4 (36.4%)	2 (10.5%)	
3	1 (9.1%)	2 (10.5%)	
4	1 (9.1%)	2 (10.5%)	
Item 3 “face hurt”			
0	3 (27.3%)	10 (52.6%)	0.73
1	3 (27.3%)	3 (15.8%)	
2	2 (18.2%)	2 (10.5%)	
3	2 (18.2%)	2 (10.5%)	
4	1 (9.1%)	2 (10.5%)	
Item 4 “blow nose”			
0	1 (9.1%)	2 (10.5%)	0.98
1	3 (27.3%)	3 (15.8%)	
2	3 (27.3%)	6 (31.6%)	
3	2 (18.2%)	4 (21.1%)	
4	2 (18.2%)	4 (21.1%)	
Item 5 “coughing”			
0	4 (36.4%)	10 (52.6%)	0.59
1	0 (0.0%)	2 (10.5%)	
2	4 (36.4%)	4 (21.1%)	
3	1 (9.1%)	1 (5.3%)	
4	1 (9.1%)	2 (10.5%)	
Item 6 “mucus in throat”			
0	1 (9.1%)	4 (21.1%)	0.92
1	4 (36.4%))	6 (31.6%)	
2	0 (0.0%)	1 (5.3%)	
3	3 (27.3%)	4 (21.1%)	
4	3 (27.3%)	4 (21.1%)	
Item 7 “mucus in nose”			0.009
0	1 (9.1%)	3 (15.8%)	
1	3 (27.3%)	0 (0.0%)	
2	2 (27.3%)	7 (36.8%)	
3	1 (9.1%)	8 (42.1%)	
4	4 (36.4%)	1 (5.3%)	
Item 8 “smell problems”			
0	7 (63.6%)	5 (26.3%)	0.08
1	1 (9.1%)	6 (31.6%)	
2	2 (18.2%)	1 (5.3%)	
3	0 (0.0%)	0 (0.0%)	
4	1 (9.1%)	7 (36.8%)	
Item 9 “awake at night”			
0	4 (36.4%)	7 (36.8%)	0.008
1	0 (0.0%)	9 (47.4%)	
2	1 (9.1%)	1 (5.3%)	
3	2 (18.2%)	0 (0.0%)	
4	4 (36.4%)	2 (10.5%)	
Item 10 “fatigued”			
0	4 (36.4%)	2 (10.5%)	0.08
1	1 (9.1%)	10 (52.6%)	
2	2 (18.2%)	2 (10.5%)	
3	3 (27.3%)	2 (10.5%)	
4	1 (9.1%)	3 (15.8%)	
Item 11 “worried will worsen”			
0	5 (45.5%)	7 (36.8%)	0.20
1	4 (36.4%)	8 (42.1%)	
2	1 (9.1%)	0 (0.0%)	
3	0 (0.0%)	4 (21.1%)	
4	1 (9.1%)	0 (0.0%)	
Item 12 “frustrated by condition”			
0	2 (18.2%)	4 (21.1%)	0.56
1	1 (9.1%)	3 (15.8%)	
2	3 (27.3%)	5 (26.3%)	
3	1 (9.1%)	5 (26.3%)	
4	4 (36.4%)	2 (10.5%)	

Values correspond to numbers (proportions) for categorical variables and means (± standard error) for quantitative variables

## Discussion

In this study, we describe the cross-cultural adaptation of a new CRS-specific patient reported outcome measure, the CRS-PRO [5]. This questionnaire reliably measures the symptoms and psychosocial impact of CRS, including its two major clinical phenotypes, CRSwNP and CRSsNP [5–7]. By following ISPOR guidelines, the patient experience was translated as accurately as possible.

### Comparison with other study

Other teams have already used the same methodology [10–13]. Some authors have highlighted major cultural differences within populations that were generally supposed to be comparable [14]. Thus, the translation-back translation process we used is essential to obtain a faithful rendering of the original document [15]. Our goal was to obtain a translation of the idea or concept, rather than a literal translation of each item.

### Strength of the study

ISPOR recommendations were chosen for the cross-cultural adaptation process, which made it possible to obtain a French culturally adapted version of the CRS-PRO equivalent to the English version [8]. ISPOR recommendations stipulate that at least two translators should perform an English‐French translation independently, followed by a coordination meeting. Therefore, we chose three independent translators to translate the concept behind the questionnaire as accurately as possible and ensure reproducibility. One major strength of this cross-cultural adaptation is using the translation‐back translation single blinded process, thus allowing the designer of the English questionnaire to correct residual misunderstandings.

### Clinical applicability of the study

As recommended by cross-cultural adaptation guidelines, it is advisable to assemble a panel of experts to test the questionnaire. During step 2, *French version 2* was submitted to a panel of six health care professionals (5 ORL and 1 pneumologist) and also 3 patients. This step, crucial in the translation of the questionnaires, led to many changes. It was essential to include a pneumologist but also non-medical professionals (herein patients) since the points of view of surgeons are sometimes biased, especially regarding the understandability of technical terms for the general population. It is recommended to include patients themselves in the translation process during step 3. Indeed, by gathering their opinion on each item, since the questionnaire is ultimately intended for them, it increases the fluidity of the questionnaire. We chose to include some patients similar to target patients immediately in step 2 and their remarks gave rise to important changes leading to *French version 3.*

The test–retest reliability was used to assess the consistency and reproducibility of results obtained from patients with French version 5 and confirmed the reproducibility and efficiency of this questionnaire although it has been translated into French language. It proves that the translation‐back translation process does not affect its validity. No translation is perfect; conceptual differences may always remain. When translated back into English, it was considered that these differences were minimal and did not lead to a change in the meaning and understanding of the questionnaire.

## Conclusion

The French version of the validated CRS-PRO questionnaire is now an adapted and well accepted instrument to evaluate the CRS symptoms and QOL in the French speaking population. This tool can be especially valuable in follow-up examinations, to measure the outcome of medical and surgical treatment of patients with CRS and for the comparison of results with the international literature and in clinical trials.

## Data Availability

Available on reasonable request.
